# Role of pepper MYB transcription factor CaDIM1 in regulation of the drought response

**DOI:** 10.3389/fpls.2022.1028392

**Published:** 2022-10-11

**Authors:** Junsub Lim, Chae Woo Lim, Sung Chul Lee

**Affiliations:** Department of Life Science (BK21 Program), Chung-Ang University, Seoul, South Korea

**Keywords:** ABA, drought, MYB, pepper, transcription factor

## Abstract

Abscisic acid (ABA) is a major phytohormone that plays important roles in stress responses, including regulation of gene expression and stomatal closure. Regulation of gene expression by transcription factors is a key cellular process for initiating defense responses to biotic and abiotic stresses. Here, using pepper (*Capsicum annuum*) leaves, we identified the MYB transcription factor *CaDIM1* (*Capsicum annuum* Drought Induced MYB 1), which was highly induced by ABA and drought stress. CaDIM1 has an MYB domain in the N-terminal region and an acidic domain in the C-terminal region, which are responsible for recognition and transactivation of the target gene, respectively. Compared to control plants, *CaDIM1*-silenced pepper plants displayed ABA-insensitive and drought-sensitive phenotypes with reduced expression of stress-responsive genes. On the other hand, overexpression of *CaDIM1* in Arabidopsis exhibited the opposite phenotypes of *CaDIM1*-silenced pepper plants, accompanied by enhanced ABA sensitivity and drought tolerance. Taken together, we demonstrate that CaDIM1 functions as a positive regulator of the drought-stress response *via* modulating ABA-mediated gene expression.

## Introduction

Since plants are sessile organisms, they have developed mechanisms that facilitate adaptation to environmental stresses, such as high salinity, extreme temperatures and drought. Drought is a major environmental stress that causes osmotic stress to cells, damages plant tissues, and limits plant development and growth, ultimately reducing agricultural crop productivity (Zhu, 2002). Plants improve their drought tolerance by adjusting physiological and molecular processes, such as stomatal aperture, stress-related gene expression, and abscisic acid (ABA) biosynthesis (Lee and Luan, 2012). ABA is a key phytohormone that is involved in responding to abiotic stress, especially drought stress. Previous studies have elucidated ABA signaling core components and their interactors, which play a role upstream or downstream of ABA signaling core components (Umezawa et al., 2010; Ng et al., 2014; Yang et al., 2017; Kumar et al., 2019). Under drought stress, endogenous ABA is synthesized and accumulated in various plant tissues (Raghavendra et al., 2010). In the cell, ABA is perceived by ABA receptors, which transfer ABA signaling to downstream proteins by inhibiting protein phosphatases, leading to the activation of protein kinases (Lim et al., 2015). Activated protein kinases promote ion channel activation and gene expression *via* phosphorylation of the SLAC1 anion channel and transcription factors, respectively (Geiger et al., 2009; Lee et al., 2009; Geiger et al., 2010).

In the drought-stress condition, various transcription factors modulate stress-related genes *via* ABA-dependent or -independent pathways (Yoshida et al., 2014; Joshi et al., 2016). Transcription factors are classified into families based on their functional and structural features, especially their DNA-binding domains (Wingender et al., 2015). In response to drought stress, the bZIP, HD-ZIP, bHLH, NAC, AP2/ERF, and MYB families are activated or inactivated, and become involved in modulating the expression of stress-related genes (Yang et al., 2010). MYB transcription factors have highly conserved N-terminal MYB DNA-binding domain repeats, named R1, R2, and R3. The major MYB transcription factors belong to the R2R3-MYB subfamily, which participates in plant development, metabolic mechanisms, and stress responses (Dubos et al., 2010; Millard et al., 2019). Arabidopsis MYB transcription factors are involved in responding to drought stress *via* modulating stomatal opening and closure (Cominelli et al., 2008; Jung et al., 2008; Oh et al., 2011). For example, *MYB60* expressed in Arabidopsis guard cells negatively modulated drought stress by regulating stomatal movement. The *myb60* mutant enhanced drought tolerance by repressing stomatal opening (Cominelli et al., 2008), whereas *MYB60*-overexpressing plants showed hypersensitivity to drought stress (Oh et al., 2011). Moreover, overexpression of *MYB44* increased ABA sensitivity and drought tolerance *via* enhancing stomatal closure (Jung et al., 2008). In addition to modulating stomatal movement, many MYB transcription factors facilitate drought tolerance by participating in ABA signaling. For example, MYB15, MYB37, and MYB96 modulate seed germination and root growth in response to ABA (Ding et al., 2009; Seo et al., 2009; Yu et al., 2016). However, in pepper plants, the biological function of MYB transcription factors in the drought-stress response has not yet been revealed.

In this study, we used RNA-seq analysis to isolate *CaDIM1* (*Capsicum annuum* Drought-Induced Myb transcription factor 1) from pepper leaves that were subjected to drought stress. *CaDIM1* expression was induced in response to drought and ABA treatments, and CaDIM1 localized in the nucleus. Moreover, we elucidated that the CaDIM1 C-terminal acidic domain contributes to transactivational activity. *CaDIM1* knockdown in pepper and *CaDIM1*-OX Arabidopsis plants showed altered phenotypes under ABA and drought stress treatments. These results indicate that CaDIM1 modulates drought tolerance *via* enhancing ABA signaling.

## Results

### Molecular characterization of CaDIM1

**Figure 1:**
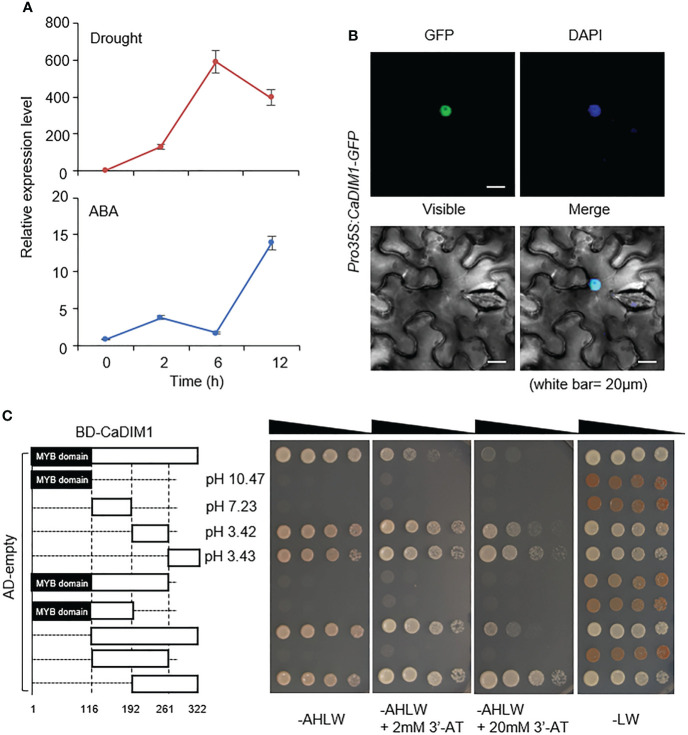
Molecular characterization of CaDIM1. **(A)** Expression levels of *CaDIM1* in the pepper leaves after treatment with abscisic acid (ABA) (100 μM), or drought. The pepper *Actin1* (*CaACT1*) was used as an internal control. **(B)** Subcellular localization of green fluorescent protein (GFP)-tagged CaDIM1 proteins. Expression of GFP in *Nicotiana benthamiana* leaves was detected under a confocal microscope. 4′,6-Diamidino-2-phenylindole (DAPI) was used as a nuclear marker. The white scale bars represent 20 μm. **(C)** Transactivation of the GAL4-responsive promoter by full-length and truncated forms of CaDIM1 fused to the GAL4 DNA-binding domain in the yeast strain AH109. Yeast harboring each construct were grown on selection media (SC-adenine-histidine-leucine-tryptophan [AHLW] SC-leucine-tryptophan [LW]). Representative images were taken at 5 days after incubation. BD, DNA-binding domain; AD, activation domain.

To identify drought-induced transcription factors in pepper, we performed RNA-seq analysis with drought-treated pepper leaves, and then isolated the pepper *CaDIM1* (*Capsicum annuum* Drought Induced Myb 1) gene corresponding to CA10g05760 and Capana10g000613 in the genomes of *Capsicum annuum* cv. CM334 (Kim et al., 2014) and cv. Zunla-1 (Qin et al., 2014), respectively. The *CaDIM1* cDNA contains an open reading frame of a 966-bp nucleic acid and encodes 321 amino acid residues. The encoded protein has an isoelectric point of 6.85 and a molecular weight of 36.49 kD. Multiple sequence alignment analysis showed a high amino acid sequence identity (53–82.6%) and similarity (64.3–88.5%) between CaDIM1 and other plant species’ Myb proteins (**Supplementary Figure S1**). The phylogenetic tree shows that CaDIM1 and its homologous protein sequences are phylogenetically distant (**Supplementary Figure S2**). In particular, we found that the deduced amino acid sequences of CaDIM1 and its pepper paralog CaMYB102A share 61.0% identity and 69.4% similarity (**Supplementary Figure S3A**). *CaDIM1* was identified in the drought-treated pepper leaves; therefore, we evaluated whether *CaDIM1* is induced by abiotic stress treatment. To determine the expression pattern of *CaDIM1* under various abiotic stress conditions, we performed qRT–PCR analysis using first and second pepper leaves after drought and ABA treatments (**Figure 1A**). Drought and ABA treatments induced *CaDIM1* transcription in pepper leaves. Drought treatment induced *CaDIM1* transcription after 2 h, with transcription reaching the maximum level at 6 h but decreasing at 12 h. On the other hand, ABA treatment induced *CaDIM1* transcription after 2 h, with transcription reaching the maximum level at 12 h. As a paralog, CaMYB102A also showed similar expression patterns, but this gene reached a peak at 2 h after ABA treatment (**Supplementary Figure S3B**). These results suggest that *CaDIM1* might be involved in abiotic stress signaling. To investigate the subcellular localization of the CaDIM1 protein, we fused the GFP reporter gene to the C-terminal of *CaDIM1* under the 35S promoter (*pro35S:CaDIM1-GFP*), and transiently expressed GFP-fused proteins in the epidermal cells of *N. benthamiana*. CaDIM1-GFP protein localized in the nucleus and generated GFP signals that overlapped with DAPI staining signals (**Figure 1B**).

CaDIM1 has an MYB domain in the N-terminal region and an acidic domain in the C-terminal region. To test whether CaDIM1 has transactivational activity and functions as a transcriptional activator, we conducted a transactivation assay in yeast using GAL4-responsive reporter system. The full-length CaDIM1 and nine truncated fragments were cloned into the pGBKT7, carrying the Gal4 DNA-binding domain, and then transformed into yeast strain AH109 with the pGADT7 (**Figure 1C**). We determined the ability of each fragment to activate transcription from UAS (upstream activation sequence), based on yeast growth in selection medium lacking Ade, Trp, His, and Leu. The full length of CaDIM1-transformed yeast grew on a selection medium, suggesting that CaDIM1 presumably functions as a transcription factor. However, truncated CaDIM1 containing the MYB domain (amino acids 1-192) had no auto activation activity. On the other hand, CaDIM1 containing the acidic domain (amino acids 193-322) showed auto activation activity in the selection medium, demonstrating that the acidic domain has control of transcriptional activity.

### Reduced tolerance of *CaDIM1*-silenced pepper plants to drought stress

**Figure 2:**
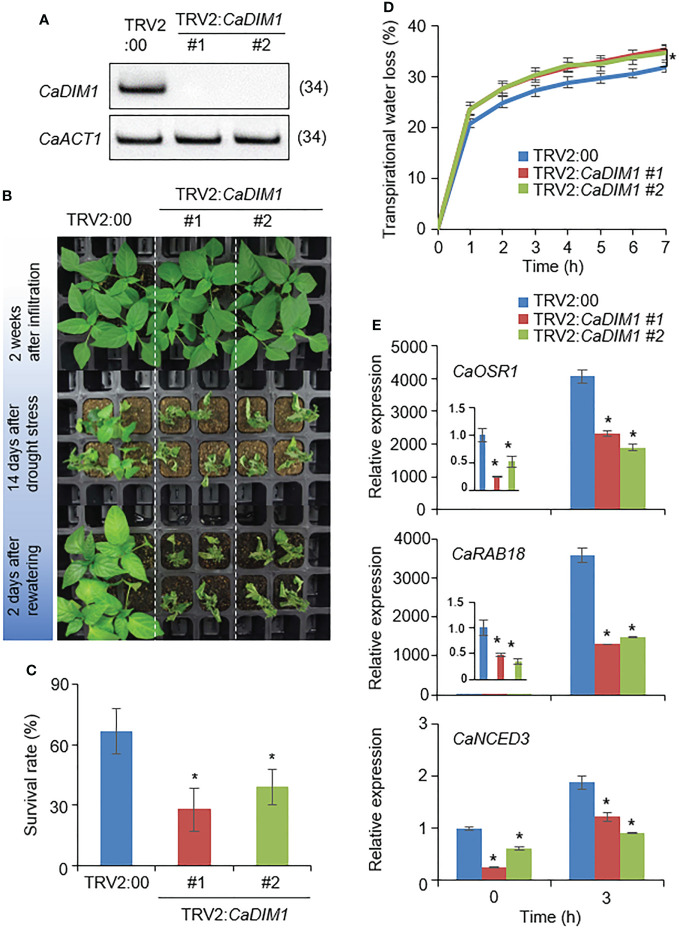
Reduced tolerance of *CaDIM1*-silenced pepper plants to drought stress. **(A)** RT-PCR assay of *CaDIM1* expression in *CaDIM1*-silenced pepper plants and control plants (TRV2:00). The pepper *Actin1* (*CaACT1*) gene was used as an internal control. **(B)** Drought sensitivity of control (TRV2:00) and *CaDIM1*-silenced pepper (TRV2:*CaDIM1*) plants. Four-week-old plants of each line were re-watered for 2 days after drought stress was imposed for 14 days by withholding watering. Representative images were taken before (top) and after (middle) drought, as well as after 2 days of re-watering (bottom). **(C)** Survival rates of TRV2:00 and TRV2:*CaDIM1* pepper plants after 2 days of re-watering. Data represent the mean ± standard error of three independent experiments; in each of these experiments, 20 plants were evaluated. **(D)** Transpirational water loss from the leaves of TRV2:00 and TRV2:*CaDIM1* plants. Leaves from plants of each line were detached, and the fresh weights of leaves were measured every hour for 7 h. Data represent the mean ± standard error of three independent experiments; in each of these experiments, 10 plants were evaluated. **(E)** Quantitative real-time polymerase chain reaction analysis of stress-responsive genes in the leaves of *CaDIM1*-silenced pepper plants (TRV2:*CaDIM1*) and control plants (TRV2:00). Control and *CaDIM1*-silenced pepper plants were subjected to dehydration for 3 h after detachment. The relative expression levels (ΔΔCT) of each gene were normalized to the geometric mean of the pepper *Actin1 (CaACT1*) gene, which was used as an internal control. Asterisks indicate significant differences between TRV2:00 and TRV2:*CaDIM1* plants (Student’s *t*-test; *P < 0.05).

To investigate the biological role of *CaDIM1* in drought-stress conditions, we performed a virus-induced gene silencing (VIGS) analysis (**Figure 2**). We generated two VIGS constructs: *CaDIM1-1* (378–677) and *CaDIM1-2* (667–966). To confirm the efficiency of the gene silencing, we performed an RT–PCR analysis, which revealed that the expression levels of *CaDIM1* were lower in *CaDIM1*-silenced pepper plants (TRV2:*CaDIM1-1*, TRV2:*CaDIM1-2*) than in control plants (TRV2:00) (**Figure 2A**). To investigate whether the reduced expression of *CaDIM1* alters drought responses, control and *CaDIM1*-silenced pepper plants were subjected to drought stress (**Figure 2**). There were no phenotypic differences between *CaDIM1*-silenced and control pepper plants under normal conditions (**Figure 2B**; upper panel). However, after drought treatment for 14 days followed by rewatering for 2 days, *CaDIM1*-silenced pepper plants showed more wilted phenotypes than control plants (**Figure 2B**; middle and lower panels). The survival rate of control plants was higher (66.67%) than that of *CaDIM1*-silenced pepper plants (27.78% and 38.89%, **Figure 2C**). To confirm whether the drought-sensitive phenotype of *CaDIM1*-silenced pepper plants was due to differences in water-retention capacity, transpirational water loss was evaluated using detached leaves from control and *CaDIM1*-silenced pepper plants (**Figure 2D**). The transpirational water loss was higher in *CaDIM1*-silenced pepper leaves than in control leaves. Previous studies have revealed that altered survival rates under drought stress are associated with the expression levels of stress-related genes (Joo et al., 2019). Therefore, we investigated whether reduced expression of *CaDIM1* have an effect on the induction of stress-related genes (**Figure 2E**). To do so, we performed qRT–PCR analysis of stress-related genes in pepper leaves from plants treated with drought stress. The expression levels of these genes, including *CaOSR1*, *CaRAB18*, and *CaNCED3*, were lower in *CaDIM1-*silenced plants than in control pepper plants.

### Reduced ABA sensitivity of *CaDIM1*-silenced pepper plants

**Figure 3:**
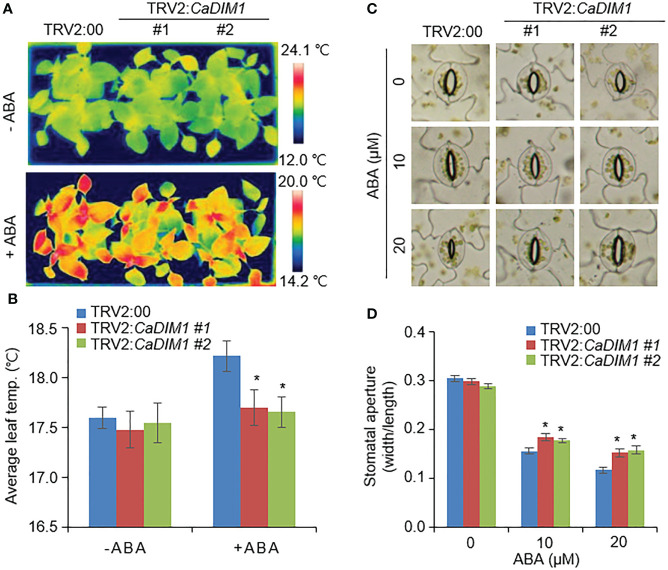
Reduction of ABA-mediated stomatal closure in the leaves of *CaDIM1*-silenced pepper plants. **(A, B)** Leaf temperatures of TRV2:00 and TRV2:*CaDIM1* plants before and after exposure to ABA. Leaves from the plants of each line were sprayed with 0 and 100 μM ABA. At 3 h after treatment, representative thermographic images were taken **(A)** and leaf temperature was measured **(B)**. **(C, D)** ABA-induced stomatal closure in TRV2:00 and TRV2:*CaDIM1* plants. Leaf peels were incubated with 0, 10, and 20 μM ABA. Representative images were taken **(C)** and the stomatal aperture of each line was measured 3 h after treatment **(D)**. All data represent the mean ± standard error of three independent experiments. Asterisks indicate significant differences between TRV2:00 and TRV2:*CaDIM1* plants (Student’s *t*-test; *P < 0.05).

Previous studies have suggested that differences in drought stress and transpiration, which ultimately affect survival rates, are caused by differences in ABA sensitivity (Lim et al., 2017). To determine ABA sensitivity, we measured leaf temperature and stomatal aperture with or without ABA treatment (**Figure 3**). There were no significant differences in leaf temperature or stomatal aperture between the *CaDIM1*-silenced pepper and control plants in the absence of ABA. ABA treated leaves exhibited higher temperatures and reduced stomatal aperture in both *CaDIM1*-silenced pepper and control plants than without ABA treatment. However, *CaDIM1*-silenced pepper plants exhibited low leaf temperatures (**Figures 3A, B**) and large stomatal aperture (**Figures 3C, D**) in comparison to control plants. These results reveal that *CaDIM1* plays a positive role in drought-stress conditions *via* modulating ABA sensitivity.

### Enhanced tolerance of *CaDIM1*-OX plants to drought stress

**Figure 4:**
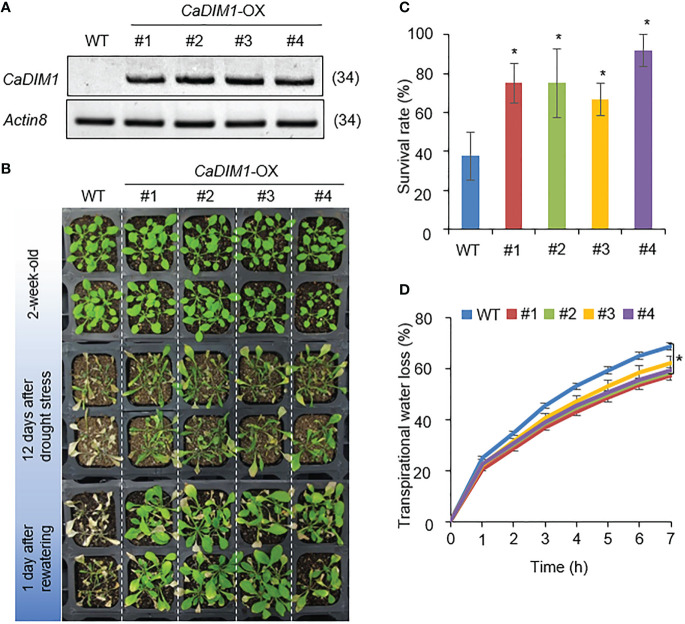
Enhanced tolerance of *CaDIM1*-overexpressing (OX) transgenic Arabidopsis plants to drought stress. **(A)** RT-PCR analysis of wild-type (WT) and *CaDIM1*-overexpressing (OX) transgenic Arabidopsis plants. The Arabidopsis *Actin8* gene was used as an internal control. **(B)** Drought-tolerant phenotype of *CaDIM1*-OX transgenic plants. Two-week-old wild-type (WT) and transgenic plants were subjected to drought stress by withholding watering for 12 days and subsequently re-watering for 1 day. The survival rate of each line was measured after re-watering. Data represent the mean ± standard error of three independent experiments; in each of these experiments, 20 plants were evaluated. **(C)** Survival rates of wild-type and transgenic plants after 1 day of re-watering. Data represent the mean ± standard error of three independent experiments; in each of these experiments, 20 plants were evaluated. **(D)** Transpirational water loss from the leaves of wild-type and transgenic plants at different time points after leaf detachment. Asterisks indicate significant differences between wild-type and *CaDIM1-*OX plants (Student’s *t*-test; *P < 0.05).

To investigate additional biological functions of *CaDIM1*, we generated Arabidopsis transgenic plants that overexpressed *CaDIM1* and obtained four independent T_3_ homozygous transgenic lines (*CaDIM1-*OX #1, #2, #3, #4) that showed high expression of *CaDIM1* (**Figure 4A**). To verify whether the enhanced expression of *CaDIM1* alters drought responses, both wild-type and *CaDIM1*-OX plants were subjected to drought stress (**Figure 2B**). All plants showed similar phenotypes under normal growth conditions (**Figure 4B**, upper panel). However, after 12 days of drought stress followed by 1 day of rewatering, *CaDIM1*-OX plants displayed less wilted phenotypes than wild-type plants (**Figure 4B**, middle and lower panels). Only 37.5% of wild-type plants resumed growth, while approximately 66–91% of *CaDIM1*-OX plants survived (**Figure 4C**). Moreover, *CaDIM1*-OX plants showed lower transpirational water loss than wild-type plants (**Figure 4D**), indicating that the enhanced drought tolerance might be attributed to altered water-retention capacity.

### Enhanced ABA sensitivity of *CaDIM1*-OX plants

**Figure 5:**
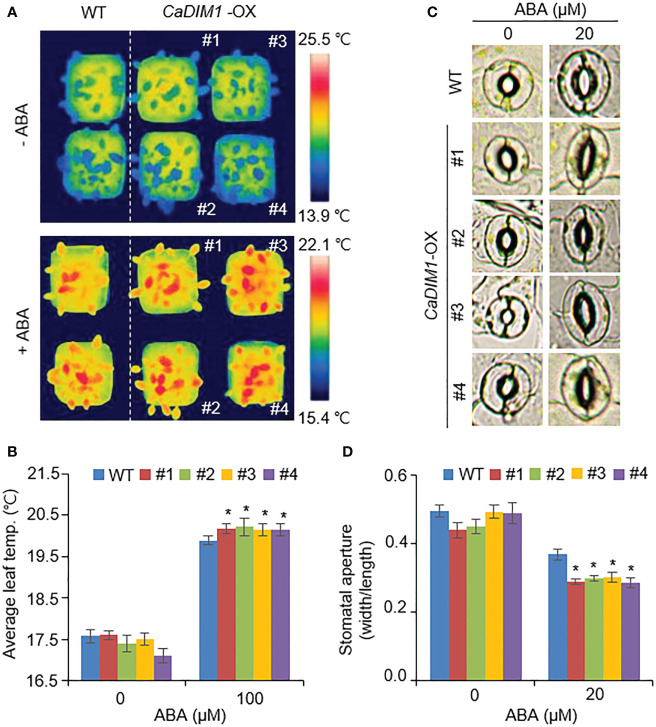
Enhanced ABA sensitivity of *CaDIM1*-overexpressing (OX) transgenic Arabidopsis plants. **(A, B)** Leaf temperatures of wild-type and *CaDIM1*-OX transgenic plants after exposure to 100 μM abscisic acid (ABA). At 6 h after treatment, representative thermographic images were taken **(A)** and leaf temperature was measured **(B)**. **(C, D)** Stomatal aperture in wild-type and *CaDIM1*-OX plants treated with ABA. Leaf peels were harvested from 3-week-old plants of each line and incubated in stomatal opening solution containing the indicated concentrations of ABA. Representative images were taken under a microscope, **(C)** and the stomatal aperture was measured **(D)**. All data represent the mean ± standard error of three independent experiments. Asterisks indicate significant differences between wild-type and *CaDIM1-*OX plants (Student’s *t*-test; *P < 0.05).

In general, drought tolerance in plants is associated with ABA sensitivity; therefore, we measured the leaf temperature and stomatal aperture in wild-type and *CaDIM1*-OX plants with or without ABA treatment (**Figures 5A–D**). Under normal conditions, leaf temperature and stomatal aperture were similar in both plants. However, under ABA treatment, *CaDIM1*-OX plants showed high leaf temperatures and reduced stomatal aperture relative to wild-type plants (**Figures 5A–D**). These results suggest that *CaDIM1* positively modulates drought stress responses by enhancing ABA sensitivity.

**Figure 6:**
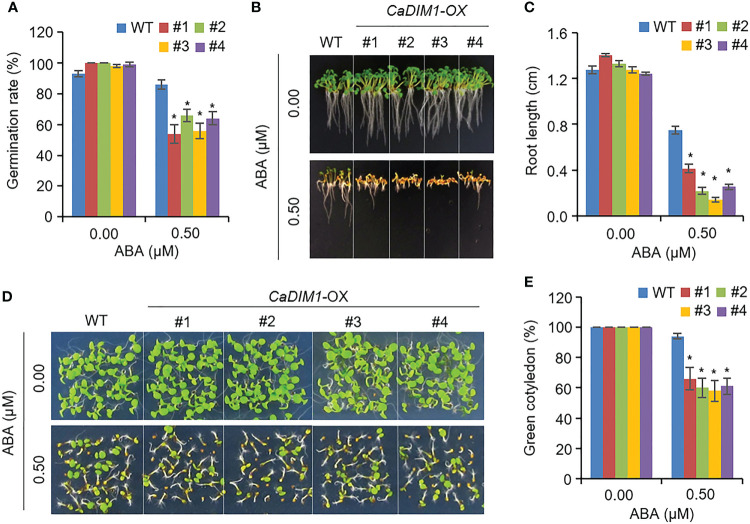
Increased sensitivity of *CaDIM1*-overexpressing (OX) transgenic Arabidopsis plants to ABA during germination and seedling growth. **(A)** Seed germination of wild-type (WT) and transgenic lines in response to ABA. Seeds were germinated on 0.5x Murashige and Skoog (MS) agar plates containing 0.0 μM and 0.5 μM of ABA. **(B, C)** Root elongation of wild-type and transgenic plants in response to 0.0 μM and 0.75 μM ABA. The representative images were taken **(B)** and the root length of each plant was measured 6 days after plating **(C)**. Data represent the mean ± standard error of three independent experiments. **(D, E)** Growth of wild-type and transgenic line seedlings on 0.5x MS agar plates containing 0.0 μM and 0.5 μM ABA. Representative images were taken **(D)** and quantification of green cotyledons was conducted 7 days after plating **(E)**. Data represent the mean ± standard error of four independent experiments; in each of these experiments, 36 seeds were evaluated. Asterisks indicate significant differences between wild-type and *CaDIM1-*OX plants (Student’s *t*-test; *P < 0.05).

ABA regulates seed germination and post-germinative growth. Therefore, we measured seed germination rates, primary root growth, and green cotyledon rate in wild-type and *CaDIM1*-OX plants (**Figure 6**). Germination rates of wild-type and *CaDIM1*-OX seeds showed no significant differences without ABA. However, when grown on an ABA-treated medium, *CaDIM1*-OX seed germination rates were lower than those of wild-type seeds (**Figure 6A**). In addition, we estimated primary root growth and green cotyledon rates in response to ABA (**Figures 6B–E**). At 10 days after plating on an ABA-treated medium, primary root growth of *CaDIM1*-OX plants was severely reduced compared with that of wild-type plants (**Figures 6B, C**). Consistent with primary root growth, cotyledon greening rate was lower in *CaDIM1*-OX plants than in wild-type plants (**Figures 6D, E**). These results suggest that enhanced *CaDIM1* expression contributed to ABA hypersensitivity in germination and post-germination growth.

## Discussion

Plants inevitably endure various environmental stresses. Drought is major stress that causes osmotic stress to plant cells, ultimately limiting growth and development. In order to survive under stressful conditions, plants have evolved defense mechanisms, controlling physiological and biochemical responses to the type of stress. Regulation of gene expression is one of the most well-known drought-stress defense mechanisms, and is modulated by transcription factors (Xiong and Zhu, 2002). The present study showed that CaDIM1 is one component of the pepper plant drought-stress response. Many regulatory proteins, including protein kinases, phosphatases, and transcription factors, have been characterized as key factors of drought stress signaling, and the involvement of these proteins in the drought-stress response is well established (Singh and Laxmi, 2015; Sah et al., 2016; Zhu, 2016). Many studies have demonstrated that transcription factors activate or repress target genes, whose expression or repression is key in response to drought stress (Dong and Liu, 2010; Yoshida et al., 2014; Xie et al., 2019). For example, the Arabidopsis ABI3 transcription factor activates *ABI5* expression, leading to drought-stress tolerance (Lopez-Molina et al., 2002). In general, DNA-binding domains of transcription factors have specific sequences and adhere to the promoter of target genes (Mitchell and Tjian, 1989; Latchman, 1990). Moreover, transcription factors contain a regulatory domain, which is rich in specific amino acids, including the acidic amino acids glutamine or proline (Licausi et al., 2013). MYB transcription factors contain two distinct domains in the N-terminal and C-terminal regions. The N-terminal domain is a conserved MYB domain for DNA binding, while the C-terminal domain is a diverse regulatory domain for activation or repression of target genes (Ambawat et al., 2013). CaDIM1 has an MYB domain and an acidic domain, which play roles in DNA binding and transactivation, respectively. However, a repression motif was not found in CaDIM1. These results indicate that CaDIM1 plays a role as a transcription activator in pepper plants.

The multiple sequence alignment with other plants showed that CaDIM1 has a conserved region in diverse plant species, and phylogenetic tree analysis showed that CaDIM1 belongs to the group 11 MYB transcription factors (**Supplementary Figures S1**, **S2**). These results suggest that CaDIM1 might have a similar function to that of other MYB transcription factors. In plants, MYB transcription factors play various roles, including UV-B tolerance and signaling, drought stress responses, ABA signaling, salt tolerance, and extreme temperature tolerance (Oh et al., 2001; Abe et al., 2003; Cominelli et al., 2005; Agarwal et al., 2006; Reyes and Chua, 2007; Jenkins, 2009; Sun et al., 2020; Yang et al., 2020). *CaDIM1* expression levels were induced in the drought-stress and ABA treatments. Generally, under drought-stress conditions, plants initiate defense processes such as biosynthesis of ABA and induction of defense-related genes (Sato et al., 2018; Takahashi et al., 2020). Due to the very low efficiency of transformation in pepper, an overexpression analysis in Arabidopsis and a VIGS assay in pepper were used for genetic analysis of *CaDIM1.* After the drought-stress treatment, *CaDIM1*-silenced pepper plants showed drought-sensitive phenotypes, which were affected by low water-retention capacity. Conversely, *CaDIM1*-OX plants showed a drought-tolerant phenotype with ABA hypersensitivity, which led to altered stomatal aperture. These genetic analyses indicate that altered expression of *CaDIM1* affects stomatal pore size, leading to regulation of water consumption and unique drought responses.

Drought-stress tolerance is related to expression levels of ABA- or stress-responsive genes (Fujita et al., 2011; Lu et al., 2019; Joo et al., 2020). ABA biosynthesis is also a key factor in determining tolerance or sensitivity (Iuchi et al., 2001). Moreover, accumulation of ABA in guard cells triggers stomatal closure, which inhibits transpirational water loss (Schroeder et al., 2001; Lim et al., 2015). In this study, the downstream target genes of CaDIM1 were not identified; however, the expression levels stress- or ABA-responsive genes, such as *CaOSR1, CaRAB18*, and *CaNCED3* were significantly lower in *CaDIM1*-silenced pepper plants than in the control pepper plants. This result demonstrates that *CaDIM1* is presumably involved in expression of these genes, directly or indirectly.

In conclusion, CaDIM1 contains an acidic domain for transactivation and is localized in the nucleus, where it may play a role as a transcriptional modulator. Silencing *CaDIM1* in pepper induced drought hypersensitivity, whereas overexpression of CaDIM1 in Arabidopsis led to drought tolerance with ABA hypersensitivity. Taken together, we demonstrated that CaDIM1 positively regulates drought-stress responses *via* modulating ABA signaling. However, we were unable to identify direct target genes and interacting partners that modulate CaDIM1 activity. Thus, further study should focus on identifying CaDIM1 direct target genes and their interacting partners, and clarifying the signaling pathway through which CaDIM1 regulates drought tolerance.

## Materials and methods

### Plant materials

Pepper (*Capsicum annuum* L., cv. Nockwang), Arabidopsis (*Arabidopsis thaliana* ecotype Col-0), and tobacco (*Nicotiana benthamiana*) seeds were sown in a mix of loam soil, sand, and compost soil (1:1:1, volume). Plants were placed in a growth chamber at 24 ± 1°C with a 16 h/8 h (light/dark) cycle. Arabidopsis seeds were germinated on MS salt with microagar (Duchefa Biochemie, Haarlem, Netherlands) and 1% sucrose.

### ABA and drought treatments

To analyze expression patterns of *CaDIM1* in pepper plants, six-leaf-stage pepper plants were treated with ABA (100 μM) or drought, as described previously (Lim et al., 2017). Pepper leaves were harvested at various time points after each treatment and were subjected to qRT–PCR assay.

To analyze drought tolerance, 4-week-old pepper and 2-week-old Arabidopsis plants were randomly organized and subjected to drought treatment by withholding watering for 14 days and 12 days, respectively. After plants were rewatered for 2 days and 1 day, respectively, the survival rate was measured. To determine drought tolerance in a quantitative manner, transpirational water loss was measured. Leaves were detached from pepper and Arabidopsis plants, and the fresh weight was measured at the various time points. The experiments were repeated three times.

### Subcellular localization analysis

To determine the subcellular location of CaDIM1, the coding region of *CaDIM1* without the stop codon was inserted to the p326GFP vector (green fluorescent protein (GFP)-fused binary vector). Green fluorescent protein (GFP)-tagged *CaDIM1* were expressed in leaves of *N. benthamiana via* agroinfiltration. A GV3101 strain of *A. tumefaciens* carrying the GFP-tagged *CaDIM1* construct with p19 was infiltrated to *N. benthamiana* leaves. The GFP and DAPI signals were detected *via* a confocal microscope.

### Transactivation assay

A transactivation assay was performed as described previously (Lim et al., 2017). The cDNA fragments of *CaDIM1* were subcloned into the pGBKT7 vector, and co-transformed into yeast strain AH109 using the lithium acetate–mediated transformation method (Ito et al., 1983). Transformant candidates were selected on SC-Leu-Trp media. Each yeast was spotted onto SC-Leu-Trp media for quantitative control or SC-Ade-His-Leu-Trp media for selection. At 5 days after incubation, the images of colony were taken.

### Virus-induced gene silencing

Virus-induced gene silencing (VIGS) was conducted to knock-down *CaDIM1* in pepper plants as described previously (Lim et al., 2017). Using the VIGS tool (http://vigs.solgenomics.net), two 300-bp fragments of the *CaDIM1* coding sequence, *CaDIM1*-1 (378–677) and *CaDIM1*-2 (667–966) with a target region score of above 99%, were designed to avoid off-target of silencing; each region was subsequently amplified by PCR. The *CaDIM1* gene fragments were inserted into the pTRV2 vector and introduced into *A. tumefaciens* strain GV3101 *via* electroporation. A GV3101 containing pTRV1, pTRV2:00, pTRV2:*CaDIM1-1*, and pTRV2:*CaDIM1-2* was infiltrated to pepper cotyledons.

### Generation of transgenic Arabidopsis plants

To generate *CaDIM1* transgenic plants in Arabidopsis, the p326GFP vector containing the coding region for *CaDIM1* without the stop codon was used. The GV3101 strain of *A. tumefaciens* containing the 35S:*CaDIM1*-GFP construct was inoculated into Arabidopsis using the floral dip method (Clough and Bent, 1998). All transgenic plants were generated on the Col-0 background. To select *CaDIM1*-OX plants, seeds were harvested from the transgenic plants and sown on MS media containing 25 μg·mL^-1^ phosphinothricin.

### Stomatal aperture bioassay and thermal imaging

Stomatal pore size measurement was performed as previously described (Lim et al., 2017). Briefly, leaf peels were placed in stomatal opening solution (SOS: 10 mM MES-KOH, 50 mM KCl, and 10 mM CaCl_2_) under the light conditions for 3 h. After incubation for 3 h, stomata closing was induced by 10 μM and 20 μM of ABA. After an additional 3 h, stomatal pore sizes were measured by microscope.

To estimate leaf temperature, first and second leaves of pepper and 3-week-old Arabidopsis plants were used. Absence or presence of 100 μM ABA, images were taken by an infrared camera (T420; FLIR systems, Wilsonville, OR, USA).

## Data availability statement

The original contributions presented in this study are included in the article/**Supplementary Material** and the data presented in this study are deposited in the NCBI repository, accession number OP382712. Further inquiries can be directed to the corresponding author.

## Author contributions

JL, CW, SL: conceptualization. JL and CW: methodology. JL and CW: data analysis. CW and SL: writing. All authors contributed to the article and approved the submitted version.

## Funding

This work was supported by a grant from the Agriculture & Technology Development (Project No. PJ01652101), and the National Research Foundation of Korea (NRF) grant funded by the Korea Government (MSIT) (2021R1A2C2006338), Rural Development Administration, Republic of Korea.

## Conflict of interest

The authors declare that the research was conducted in the absence of any commercial or financial relationships that could be construed as a potential conflict of interest.

## Publisher’s note

All claims expressed in this article are solely those of the authors and do not necessarily represent those of their affiliated organizations, or those of the publisher, the editors and the reviewers. Any product that may be evaluated in this article, or claim that may be made by its manufacturer, is not guaranteed or endorsed by the publisher.

## Supplementary material

Supplementary Figure 1 Alignment of the deduced CaDIM1 amino acid sequence with those of Solanum lycopersicum (accession no. XP_004248359.1), Solanum tuberosum (accession no. XP_006352596.1), Nicotiana tabacum (accession no. XP_016436111.1), and Arabidopsis thaliana (accession no. NP_567626.1) proteins. Identical amino acid residues are highlighted in black. Multiple alignment of the CaDIM1 protein sequence and its homologous proteins was performed using ClustalW2.

Supplementary Figure 2 Phylogenetic tree analysis of the CaDIM1protein. A BLAST search was performed by using the deduced CaDIM1 and CaMYB102A amino acid sequences with highest similarity gathered from Arabidopsis. Multiple sequence alignment was performed using Clustal Omega (http://www.ebi.ac.uk/Tools/msa/clustalo/) with the default settings. The phylogenetic tree was drawn using the neighbor-joining method with MEGA software (version 7.0). Bootstrap values were calculated from 1,000 bootstrap replications and are indicated at each branch point. The scale bar indicates genetic distance.

Supplementary Figure 3 Characterization of CaMYB102A, a paralog of CaDIM1. **(A)** Pairwise sequence alignment of CaMYB102A and CaDIM1. Sequence alignment was performed using EMBOSS Needle (https://www.ebi.ac.uk/Tools/psa/emboss_needle/index.html) with the default settings. **(B)** Expression levels of CaMYB102A gene in pepper leaves after treatment with abscisic acid (ABA) (100 μM) or drought. The pepper Actin1 (CaACT1) was used as an internal control. **(C)** Expression levels of CaDIM1 and CaMYB102A gene in the leaves of CaDIM1-silenced pepper plants treated with drought stress for 6 h. After quantitative real time polymerase chain reaction analysis RT-PCR analysis, the relative expression levels (ΔΔCT) of each gene were normalized using the pepper Actin1 (CaACT1) gene as an internal control and the expression value of each gene in TRV2:00 was set as 1.0. Asterisks indicate significant differences between TRV2:00 and TRV2:CaDIM1 plants (Student’s t-test; *P < 0.05).

Supplementary Table 1 Sequences of primers used in this study.
