# Supplementary material for: Role of pepper MYB transcription factor CaDIM1 in regulation of the drought response
Source: Front Plant Sci. 2022 Oct 11;13:1028392. doi: 10.3389/fpls.2022.1028392 (PMC9592997; doi:10.3389/fpls.2022.1028392)
Supplement: Supplementary Figure 1 — Alignment of the deduced CaDIM1 amino acid sequence with those of Solanum lycopersicum (accession no. XP_004248359.1), Solanum tuberosum (accession no. XP_006352596.1), Nicotiana tabacum (accession no. XP_016436111.1), and Arabidopsis thaliana (accession no. NP_567626.1) proteins. Identical amino acid residues are highlighted in black. Multiple alignment of the CaDIM1 protein sequence and its homologous proteins was performed using ClustalW2. [file Image_1.pdf]

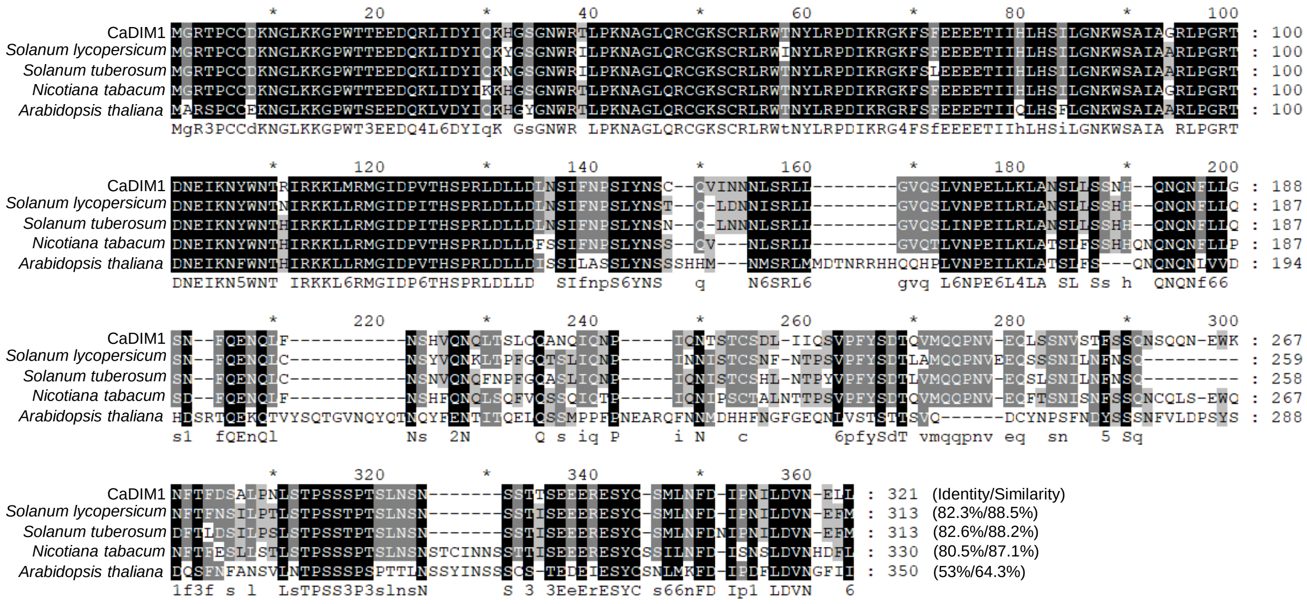

Supplementary Figure 1. Alignment of the deduced CaDIM1 amino acid sequence with those of *Solanum lycopersicum* (accession no. XP_004248359.1), *Solanum tuberosum* (accession no. XP_006352596.1), *Nicotiana tabacum* (accession no. XP_016436111.1), and *Arabidopsis thaliana* (accession no. NP_567626.1) proteins. Identical amino acid residues are highlighted in black. Multiple alignment of the CaDIM1 protein sequence and its homologous proteins was performed using ClustalW2.
