# Supplementary material for: Role of pepper MYB transcription factor CaDIM1 in regulation of the drought response
Source: Front Plant Sci. 2022 Oct 11;13:1028392. doi: 10.3389/fpls.2022.1028392 (PMC9592997; doi:10.3389/fpls.2022.1028392)
Supplement: Supplementary Table 1 — Sequences of primers used in this study. [file Table_1.pdf]

Supplemental Table S1. Sequences of primers used in this study

Primer name	Primer sequence (5'-3')
For cloning	
CaDIM1	Forward: ATGGGAAGAACACCTTGTTGTGACA
(CA10g05760)	Reverse: TTACAAAAGTTCATTAACATCCAAAATA
CaDIM1 1-116	Forward: ATGGGAAGAACACCTTGTTGTGACA
	Reverse: TCAAAGTTTTTTCCGAATGCGA
CaDIM1 117-192	Forward: ATGAGGATGGGAATTGATCCAGTG
	Reverse: TCATTGAAAATTACTTCCCAACAAGA
CaDIM1 193-261	Forward: ATGGAAAATCAACTATTCAATTCCC
	Reverse: TCAGCTATTTTGCGAGCTAAAG
CaDIM1 262-322	Forward: ATGCAACAAAATGAGTGGAAAAAT
	Reverse: TTACAAAAGTTCATTAACATCCAAAAT
w/o stop codon	Forward: GGGTCGAATTCGCCCTTCAAAAGTTCATTAACATCCAAAATAT
	Reverse: ATATTTTGGATGTTAATGAACTTTTGAAGGGCGAATTCGACCC
For RT-PCR	
CaDIM1	Forward: AATTCCCATGTCCAAAACCA
(CA10g05760)	Reverse: AATTTGGCAAAGCAGAATCG
CaACT1	Forward: GACGTGACCTAACTGATAACCTGAT
(CA12g08730)	Reverse: CTCTCAGCACCAATGGTAATAACTT
CaOSR1	Forward: ATGGAGGCACAACTGCACCGTC
(CA03g17780)	Reverse: GGCCCACCATGAACTTCTGCAC
CaRAB18	Forward: ATGTCGCACTACGAGAACCAATATAG
(CA02g22060)	Reverse: ATCATCCTCAGAGCTGCTGGAGC
CaNCED3	Forward: TTAAGGATCTTAAGCGTGGTTATGT
(CA08g03620)	Reverse: AGATTAGTTCAAGAACGTGAATTGG
AtActin8	Forward: CAACTATGTTCTCAGGTATTGCAGA
(At1g49240)	Reverse: GTCATGGAAACGATGTCTCTTTAGT
For VIGS	
XbaI-CaDIM1-1	Forward: TCTAGATAGTCCTCGTCTTGATCTTCTTG
XhoI-CaDIM1-1	Reverse: CTCGAGTCGGAGCAGGTAGAAGTATT
XbaI-CaDIM1-2	Forward: TCTAGAACCTGCTCCGATTTAAATATTCA
XhoI-CaDIM1-2	Reverse: CTCGAGTTACAAAAGTTCATTCATAACATCC
