# Supplementary material for: Role of pepper MYB transcription factor CaDIM1 in regulation of the drought response
Source: Front Plant Sci. 2022 Oct 11;13:1028392. doi: 10.3389/fpls.2022.1028392 (PMC9592997; doi:10.3389/fpls.2022.1028392)
Supplement: Supplementary Figure 2 — Phylogenetic tree analysis of the CaDIM1protein. A BLAST search was performed by using the deduced CaDIM1 and CaMYB102A amino acid sequences with highest similarity gathered from Arabidopsis. Multiple sequence alignment was performed using Clustal Omega (http://www.ebi.ac.uk/Tools/msa/clustalo/) with the default settings. The phylogenetic tree was drawn using the neighbor-joining method with MEGA software (version 7.0). Bootstrap values were calculated from 1,000 bootstrap replications and are indicated at each branch point. The scale bar indicates genetic distance. [file Image_2.pdf]

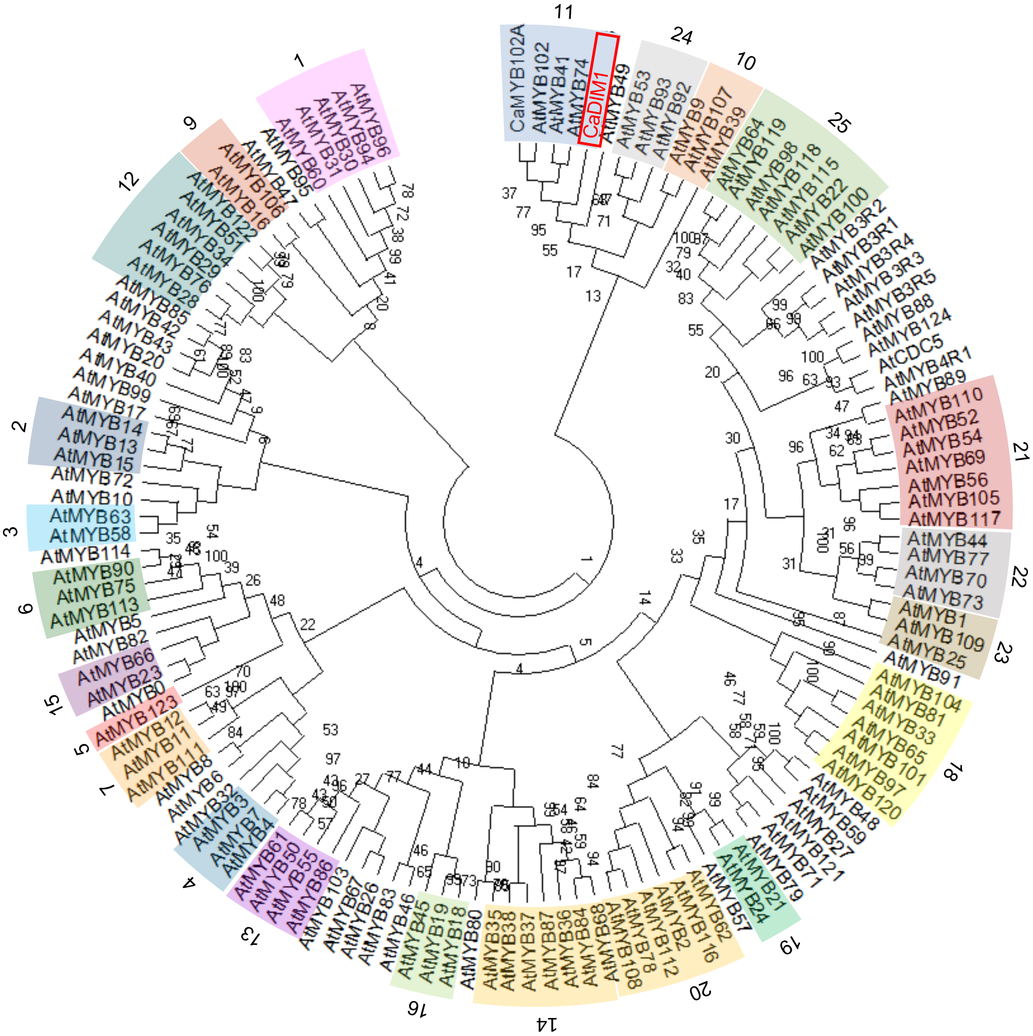

Supplementary Figure 2. Phylogenetic tree analysis of the CaDIM1protein. A BLAST search was performed by using the deduced CaDIM1 and CaMYB102A amino acid sequences with highest similarity gathered from Arabidopsis. Multiple sequence alignment was performed using Clustal Omega (http://www.ebi.ac.uk/Tools/msa/clustalo/) with the default settings. The phylogenetic tree was drawn using the neighbor-joining method with MEGA software (version 7.0). Bootstrap values were calculated from 1,000 bootstrap replications and are indicated at each branch point. The scale bar indicates genetic distance.
