# Supplementary material for: Role of pepper MYB transcription factor CaDIM1 in regulation of the drought response
Source: Front Plant Sci. 2022 Oct 11;13:1028392. doi: 10.3389/fpls.2022.1028392 (PMC9592997; doi:10.3389/fpls.2022.1028392)
Supplement: Supplementary Figure 3 — Characterization of CaMYB102A, a paralog of CaDIM1. (A) Pairwise sequence alignment of CaMYB102A and CaDIM1. Sequence alignment was performed using EMBOSS Needle (https://www.ebi.ac.uk/Tools/psa/emboss_needle/index.html) with the default settings. (B) Expression levels of CaMYB102A gene in pepper leaves after treatment with abscisic acid (ABA) (100 μM) or drought. The pepper Actin1 (CaACT1) was used as an internal control. (C) Expression levels of CaDIM1 and CaMYB102A gene in the leaves of CaDIM1-silenced pepper plants treated with drought stress for 6 h. After quantitative real time polymerase chain reaction analysis RT-PCR analysis, the relative expression levels (ΔΔCT) of each gene were normalized using the pepper Actin1 (CaACT1) gene as an internal control and the expression value of each gene in TRV2:00 was set as 1.0. Asterisks indicate significant differences between TRV2:00 and TRV2:CaDIM1 plants (Student’s t-test; *P < 0.05). [file Image_3.pdf]

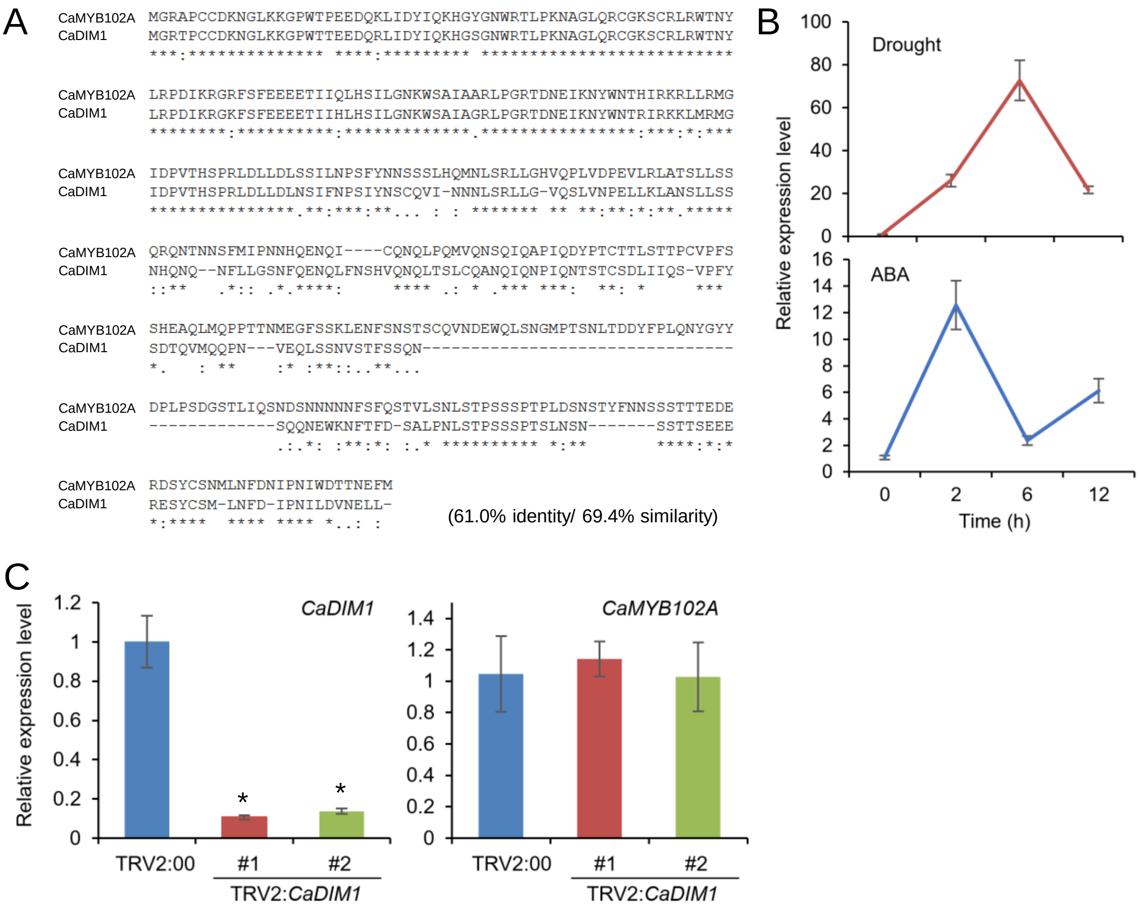


**Supplementary Figure 3.** Characterization of CaMYB102A, a paralog of CaDIM1. (A) Pairwise sequence alignment of CaMYB102A and CaDIM1. Sequence alignment was performed using EMBOSS Needle (https://www.ebi.ac.uk/Tools/psa/emboss_needle/index.html) with the default settings. (B) Expression levels of *CaMYB102A* gene in pepper leaves after treatment with abscisic acid (ABA) (100 μM) or drought. The pepper Actin1 (*CaACT1*) was used as an internal control. (C) Expression levels of *CaDIM1* and *CaMYB102A* gene in the leaves of CaDIM1-silenced pepper plants treated with drought stress for 6 h. After quantitative real time polymerase chain reaction analysis RT-PCR analysis, the relative expression levels (ΔΔCT) of each gene were normalized using the pepper Actin1 (*CaACT1*) gene as an internal control and the expression value of each gene in TRV2:00 was set as 1.0. Asterisks indicate significant differences between TRV2:00 and TRV2:*CaDIM1* plants (Student's *t*-test; * $P < 0.05$).
